# The Effect of Multilevel Surgery for Obstructive Sleep Apnea on Fatigue, Stress and Resilience

**DOI:** 10.3390/jcm12196282

**Published:** 2023-09-29

**Authors:** Su Young Jung, Young Min Mun, Gyu Man Lee, Sung Wan Kim

**Affiliations:** 1 Department of Otorhinolaryngology-Head and Neck Surgery, Myongji Hospital, Hanyang University Medical Center, Goyang 10475, Republic of Korea; 2 Department of Otolaryngology-Head and Neck Surgery, School of Medicine, Kyung Hee University, Seoul 02447, Republic of Korea

**Keywords:** obstructive sleep apnea, sleep surgery, fatigue, stress, resilience

## Abstract

Objective: To evaluate the effects of surgical treatment on fatigue, stress, and resilience in patients with obstructive sleep apnea (OSA). Methods: Sixty patients who underwent multilevel sleep surgery for OSA (OSA group) and 32 non-OSA participants (control group) were recruited at a university hospital in Korea between January 2020 and March 2022. Fatigue, stress, and resilience levels were evaluated in both groups using the Chalder fatigue scale (CFS), daily hassles scale revised (DHS-R), and Connor–Davidson resilience scale (CD-RISC), respectively. The scores of each group were compared before and 6 months after surgery. Results: The initial CFS and DHS-R scores were significantly higher, while the CD-RISC score was significantly lower, in the OSA group than in the control group (*p* < 0.05). In the patients with OSA, all three scores significantly improved after surgery (*p* < 0.05). Additionally, when compared between the groups at 6 months, there were no differences in the CFS, DHS-R, or CD-RISC scores (*p* > 0.05). Even when the OSA group was divided into a success group and a failure group according to surgical outcomes and compared with the control group, the three scores of both groups did not show statistical differences from the control group (*p* > 0.05). Conclusions: Multilevel surgery may reduce fatigue as well as stress and increase resilience in patients with OSA to levels similar to those in non-OSA individuals.

## 1. Introduction

Obstructive sleep apnea (OSA) is a chronic disease characterized by the recurrent collapse of the upper airway during sleep. Severe hypoxemia and repetitive arousals during sleep can lead to various health problems [1]. OSA has frequently been associated with several comorbidities, including metabolic, cardiovascular, renal, pulmonary, and neuropsychiatric conditions. Psychological dysfunctions associated with OSA include anxiety, depression, chronic fatigue, and a lack of energy [2]. Patients with OSA frequently complain of both fatigue and sleepiness [2,3,4,5]. Fatigue is a socially and culturally influenced state that is ill-defined; it is physical and psychological in nature, and is characterized by tiredness, decreased strength, a lack of energy, lethargy, and concentration difficulties [4]. Stress is a physical, mental, or emotional response associated with bodily or mental tension that is also commonly observed in OSA patients. Stressors can be external (environmental, psychological, or social) or internal (associated with illness or a medical procedure) and can initiate the “fight or flight” response, a complex reaction involving the nervous and endocrine systems. Moreover, stress can cause or influence the course of many medical conditions, including depression and anxiety [6]. Resilience is a measure of an individual’s ability to cope with stress and is a dynamic process of positive adaptation in the face of significant adversity or trauma. Resilient individuals display a comprehensive ability to adapt to various work and social situations as well as psychological and physical health states. Psychological resilience reportedly predicts an individual’s physiological response to stress; resilient individuals can use positive emotions to “bounce back” from stressful encounters. Moreover, resilience may act as a protective factor against the development of depression and other psychiatric disorders [7,8].

People with chronic diseases, including those with OSA, have high levels of fatigue as well as stress and low resilience [7,9,10]. Continuous positive airway pressure (CPAP) treatment, which is considered the gold standard of treatment for OSA, improves these problems [11]; however, no reports exist on the effects of sleep surgery, another effective and widely used therapeutic option, on stress, fatigue, or resilience in patients with OSA.

In this study, we compared the preoperative levels of fatigue, stress, and resilience between patients with OSA and control participants and determined whether multilevel surgery, such as uvulopalatal flap (UPF), tonsillectomy, and tongue base reduction with radiofrequency (TBR c RF), reduced these levels in the patients with OSA compared with those in the control individuals.

## 2. Materials and Methods

### 2.1. Subjects

From January 2020 to March 2022 we enrolled participants in this prospective study. Of the 97 patients who underwent sleep surgery during the period at our university hospital, 37 were excluded because they had already tried to treat their sleep problems. Consequently, 60 OSA patients were diagnosed with OSA (apnea–hypopnea index (AHI) ≥ 5) according to the results of overnight polysomnography (PSG) and underwent multilevel sleep surgery (OSA group). All patients were observed to have a tonsil size of 3 or 4 and a Friedman palatal position of III or IV upon a preoperative physical examination, which corresponded to Friedman stage II. In addition, a drug-induced sleep endoscope (DISE) showed partial/complete obstruction in the anteroposterior or lateral direction at the velum level, the lateral direction at the oropharynx level, and the anteroposterior direction at the tongue base level. Therefore, multilevel sleep surgeries, these being UPF, tonsillectomy, and TBR c RF, were performed for all of these patients. No or mild nasal septal deviation was observed upon a preoperative physical examination and PNS CT, so nasal surgery was not performed.

For comparison, patients who visited our outpatient clinic because of other otorhinolaryngological problems were recruited as controls. Individuals who did not exhibit sleep apnea, snoring, or other sleep-related symptoms were asked to complete the STOP-Bang (snoring, tiredness, observed apnea, high blood pressure, body mass index, age, neck circumference, and male gender) questionnaire for screening purposes [12]. Only those with a STOP-Bang score of >3 underwent overnight PSG. Subsequently, only patients with an AHI of <5 were included as controls. In both groups, individuals were excluded from the study if they (1) were <19 or >65 years old, (2) had previously been diagnosed with or treated for OSA, or (3) had an active medical, neurological, or psychiatric disorder that could impact stress, fatigue, or resilience. Data on basic demographic variables, medical comorbidities, and medications were obtained from the questionnaires and the patients’ medical records.

The purpose of the study was explained to the patients and each patient provided written informed consent prior to participation in the study. The study conforms to the tenets of the Declaration of Helsinki and the protocol was approved by the institutional review board of our hospital.

### 2.2. Evaluation of Fatigue, Stress, and Resilience

In this study, three surveys were used to evaluate fatigue, stress, and resilience.

The Chalder fatigue scale (CFS) was used to evaluate fatigue [13]. The CFS is a brief instrument that is frequently used in research and clinical assessment. This 14-item scale was developed by Chalder et al. and comprises questions related to physical as well as mental fatigue. Each item was scored using a 4-point Likert scale: 1 = “better than usual”, 2 = “no more than usual”, 3 = “worse than usual”, and 4 = “much worse than usual”. Higher scores indicate higher levels of fatigue and more severe subjective symptoms.

Second, the daily hassles scale revised (DHS-R) was used to evaluate stress [14]. The DHSR comprises 51 questions, each rated on a 4-point scale ranging from 1 (“not at all part of my life”) to 4 (“very much part of my life”). It incorporates problems related to work, social relations, finance, time, acceptance, and stigma associated with disease. The total score is calculated by summing the scores of all 51 questions, and ranges from 51 to 204. In the total and subscale scores, a higher score represents greater exposure to daily hassles. There are no norms for this scale in the healthy population.

Third, the Connor–Davidson resilience scale (CD-RISC) was used to evaluate resilience [15]. In this study, we used the Korean version of the CD-RISC to assess the participants. This scale consists of 25 items, each of which was rated by respondents on a 5-point scale (0 = “not true at all” to 4 = “true nearly all of the time”) according to the extent to which they believed each item applied to them over the course of the previous month. The total score is calculated by summing the scores of each item and ranges from 0 to 100. Higher scores reflect greater resilience and an elevated ability to adapt to environmental changes, stress, and fatigue.

The OSA group responded to the questionnaires before and 6 months after surgery. Similarly, the control group completed the questionnaires at the time of enrollment in the study and after 6 months. Between groups, the scores at the same time points (0 and 6 months) were compared, while those at the two different time points were compared within each group.

### 2.3. Criteria for the Evaluation of Surgical Success Rate in the OSA Group

In the OSA group, follow-up PSG was performed 6 months after surgery. According to these results, the success of the surgery was determined. The surgical success was defined according to the Sher criteria: AHI < 20/h with ≥50% AHI improvement [16].

### 2.4. Statistical Analyses

All of the collected data were entered into a single database. SPSS Version 20.0 for Windows (IBM, Armonk, NY, USA) was used for all statistical analyses. Student’s *t*-tests were conducted to compare the pre- and postoperative fatigue, stress, and resilience levels between the OSA and control groups at each time point. In addition, paired *t*-tests were used to compare the pre- and postsurgical findings within the OSA group.

A *p*-value of <0.05 was considered statistically significant for all analyses.

## 3. Results

The mean age, mean body mass index, sex ratio, marital status, and employment status did not differ significantly between the OSA and control participants. However, the PSG parameters showed significant differences between the two groups, as expected (Table 1).

When the OSA and control groups were compared at the initial time point, significant differences in the CFS, DHS-R, and CD-RISC scores were identified (Table 2).

As shown in Table 3, compared with the scores before surgery, CFS, DHS-R, and CD-RISC scores as well as PSG parameters significantly improved after surgery in patients with OSA.

In addition, when the OSA and control groups were compared at 6 months, there were significant differences in PSG parameters, but no differences in CFS, DHS-R, and CD-RISC scores between the two groups (Table 4).

When the OSA group was divided into the success group and the failure group according to the success of the surgery, there were 43 patients in the success group and 17 patients in the failure group, showing a success rate of about 71.8% in this study. Additionally, the results of the success group and failure group were compared with those of the control group. When the same analysis was performed, the CFS, DHS-R, and CD-RISC scores at 6 months after surgery in the success group showed no significant difference from the control group. The postoperative PSG parameters of the success group showed significant differences from the control group, except for LsaO2 (Table 5). The failure group showed poor outcomes on PSG parameters compared to the control group, similar to the preoperative results of the OSA group. However, the CFS, DHS-R, and CD-RISC scores did not show statistical differences from the control group, similar to the success group (Table 6).

## 4. Discussion

As a chronic disease with a lifelong course, OSA is associated with a great deal of psychological dysfunction [2,3,4,5,6,15]. The fact that CPAP therapy, which is the preferred OSA treatment, must be continually used throughout a patient’s life can cause even more psychological distress [3,11]. Several studies have examined the association between psychosocial factors and disease course in patients with OSA [2,3,4,5,6]; however, no comprehensive study has investigated the effects of surgical treatment on fatigue, stress, or resilience in patients with OSA.

Fatigue encompasses complex interactions between biological, psychosocial, and behavioral processes. It has been medically defined as a state following a period of mental or bodily activity that is characterized by a reduced capacity for work and efficiency of accomplishment, usually accompanied by a feeling of weariness, sleepiness, or irritability [4,5,9,13]. Chronic fatigue is a common complaint among patients with a variety of chronic diseases, including OSA, and is often rated as a major factor in reducing quality of life [4,9,13,17]. Several studies have reported that good adherence to PAP therapy improves symptoms such as fatigue, tiredness, and lack of energy in patients with OSA [18]. In our study, we found that multilevel surgery improved the fatigue levels of OSA patients to levels similar to those of the normal control group, which may provide evidence to support the idea that multilevel surgery may have similar efficacy to PAP therapy.

Stress refers to the general wear and tear of the body due to psycho-physiological changes that occur when an individual experiences a strong emotional response to a situation [6]. The damage caused by sleep fragmentation in OSA can manifest emotionally, including stress [19]. A correlation between cortisol levels and sleep was identified in a study on sleep restriction (4 h/night for six consecutive nights), which led to increased cortisol levels in healthy individuals [20]. Correspondingly, nocturnal awakenings in OSA have been associated with alterations in the hypothalamic–pituitary–adrenal axis, and intermittent hypoxia as well as sleep fragmentation and deprivation are thought to induce the release of cortisol. Consequently, patients with OSA may exhibit high stress levels [21,22]. In the present study, the OSA group had a higher DHS-R score than that in the control group. Moreover, the DHS-R score decreased significantly after surgery in patients with OSA. These results support previous findings that stress was significantly reduced in patients who received positive airway pressure treatment [15]. Therefore, our findings suggest that sleep surgery can improve the psychological status of patients with OSA in a manner similar to that of CPAP therapy. Bothering symptoms such as nocturia, which are common in OSA patients, are known to reduce sleep quality due to frequent awakenings during sleep [23]. This leads to an increase in stress or fatigue, resulting in a mental health burden [24]; however, our study did not investigate how patients’ individual OSA-related bothering symptoms improved. Therefore, our results cannot clarify the interrelationship between improvement in stress and improvement in bothersome symptoms of OSA patients, which should be further investigated in future follow-up studies. Resilience describes an individual’s capacity to respond positively to adverse situations, even when these pose a potential risk to their health or development [25].

Resilience has been defined as a dynamic process involving the following three key aspects: (1) at-risk individuals show better results than expected; (2) individuals adapt positively despite the occurrence of stressful experiences; and (3) individuals recover well from trauma [26]. Resilience encompasses resistance to experiences of environmental risk, stress, and adversity, and is related to individual differences in people’s stress responses [27]. In the context of chronic diseases, psychological processes such as stress can interfere with immune system functioning, thus increasing the body’s vulnerability to illness and promoting the development of symptoms [28,29]. As such, resilience is a significant psychological factor that plays a role in overcoming stress and fatigue. In the present study, patients with OSA exhibited markedly reduced resilience compared with that in the control group, suggesting that these individuals have an impaired ability to adapt to stress and fatigue. Compared with their scores before surgery, individuals in the OSA group exhibited significantly increased postoperative CDRISC scores. These results indicate that sleep surgery may improve resilience in patients with OSA.

When compared at 6 months after surgery and before surgery, the PSG parameter of the OSA group was significantly improved; however, the postoperative PSG results of the OSA group were significantly worse compared to the control group. Nevertheless, there was no significant difference in the CFS, DHS-R, or CD-RISC scores of the OSA group and the control group at 6 months. This finding demonstrates that, following sleep surgery, OSA patients exhibited similar levels of fatigue, stress, and resilience to participants in the control group, even though they did not show normalization in PSG parameters. When the results of the success group and the failure group based on Shigel’s criteria were compared with the control group, the improvement in the CFS, DHS-R, or CD-RISC scores and PSG parameters was more pronounced in the success group. It can be assumed that the operation successfully controlled the symptoms of OSA, and, as a result, each psychological symptom was improved. In the failure group there were significant poor outcomes compared to the control group in terms of the PSG parameters; however, similar to the success group, the CFS, DHS-R, or CD-RISC scores did not show statistical differences from the control group. This leads us to speculate that surgical treatment may act as a positive factor in improving a patient’s psychological symptoms, apart from the criteria for determining the success of surgery based on the PSG parameters. Previous studies have reported that 3 weeks of CPAP therapy significantly reduced fatigue and stress in patients with OSA [30,31,32]. Similarly, our study suggests that sleep surgery may reduce stress and fatigue, as well as increase resilience in OSA patients. Given these psychological benefits, surgery should be considered when determining the appropriate treatment for OSA.

Several limitations should be taken into account when interpreting the results of our study. First, the sample size was small; therefore, the results may not be representative of all patients with OSA. Second, the control group in this study was simply participants without sleep problems. Therefore, they did not undergo any surgery, so it is difficult to control the placebo effect via surgery in control group. In addition, because the control group was not OSA patients who received a sham intervention, the possibility that the improvement in the patient group was due to the observer effect (or Hawthorne effect) rather than the efficacy of the surgery itself cannot be completely ruled out. Third, the underlying causes of heightened fatigue as well as stress and low resilience in the OSA group were not elucidated. Additionally, psychological status was only evaluated based on the results of questionnaires. Finally, only short-term outcomes were studied. Based on these limitations, it would be beneficial to determine the long-term effects of adverse psychological symptoms and resilience in follow-up studies.

## 5. Conclusions

Patients with OSA exhibit increased fatigue as well as stress and reduced resilience compared with the levels observed in control individuals. The results of this study demonstrate that surgery may effectively reduce stress as well as fatigue and improve resilience in patients with OSA.

## Figures and Tables

**Table 1:** Demographic and clinical characteristics of the obstructive sleep apnea group and control groups.

	OSA Group (*n* = 60)	Control Group (*n* = 32)	Absolute Difference	95% CI	*p*-Value
Age, y, mean ± SD	42.11 ± 13.89	38.10 ± 13.02	4.01	−1.90 to 9.92	0.075
Range	19 to 77	19 to 71			
Median	40	39			
BMI, kg/m^2^, mean ± SD	26.18 ± 3.52	25.29 ± 7.14	0.89	−1.31 to 3.09	0.061
Range	19.9 to 35.9	18.7 to 32.3			
Median	25.6	24.8			
Sex (female), *n*, %	9 (15.0)	4 (12.5)	2.50	−16.47 to 17.22	0.924
Married or coupled, *n*, %	53 (88.3)	26 (81.3)	7.08	−8.76 to 26.48	0.074
Employed, *n*, %	47 (78.3)	24 (75.0)	3.33	−14.87 to 24.22	0.648
PSG parameters					
AHI, /h, mean ± SD	40.75 ± 21.38	1.28 ± 0.98	39.47	31.94 to 47.00	<0.001
RDI, /h, mean ± SD	42.91 ± 20.51	2.30 ± 1.51	40.61	33.38 to 47.84	<0.001
ODI, /h, mean ± SD	30.04 ± 20.82	1.46 ± 1.22	28.58	21.24 to 35.92	<0.001
LSaO_2_, %, mean ± SD	81.40 ± 8.18	91.75 ± 2.70	10.35	7.39 to 13.31	0.011

OSA, obstructive sleep apnea; CI, confidence interval; SD, standard deviation; BMI, body mass index; PSG, polysomnography; AHI, apnea–hypopnea index; RDI, respiratory distress index; ODI, oxygen desaturation index; and LSaO_2_, lowest oxygen saturation.

**Table 2:** Differences in the CFS, DHS-R, and CD-RISC scores between the preoperative obstructive sleep apnea group and control groups.

	OSA Group (*n* = 60)	Control Group (*n* = 32)	Mean Difference	95% CI	*p*-Value
CFS score, mean ± SD	31.50 ± 7.66	24.38 ± 4.61	7.12	4.18 to 10.06	<0.001
Range	15 to 54	20 to 38			
Median	30	28			
DHS-R score, mean ± SD	77.96 ± 21.11	67.50 ± 16.09	10.46	1.97 to 18.95	<0.001
Range	51 to 198	51 to 111			
Median	109	81			
CD-RISC score, mean ± SD	64.91 ± 16.05	71.98 ± 12.35	7.07	0.60 to 13.54	0.010
Range	12 to 100	46 to 100			
Median	56	73			

OSA, obstructive sleep apnea; CI, confidence interval; CFS, Chalder fatigue scale; SD, standard deviation; DHS-R, daily hassles scale revised; and CD-RISC, Connor–Davidson resilience scale.

**Table 3:** Changes in the CFS, DHS-R, and CD-RISC scores as well as PSG parameters before and after surgery in the obstructive sleep apnea group.

	Preoperative Score	Postoperative Score	Mean Difference	95% CI	*p*-Value
CFS score, mean ± SD	31.50 ± 7.66	25.32 ± 7.34	6.18	3.47 to 8.89	<0.001
Range	15 to 54	14 to 53			
Median	30	25			
DHS-R score, mean ± SD	77.96 ± 21.11	70.67 ± 18.25	7.29	0.56 to 14.42	0.003
Range	51 to 198	51 to 150			
Median	109	71			
CD-RISC score, mean ± SD	64.91 ± 16.05	72.14 ± 19.41	7.23	0.79 to 13.67	0.015
Range	12 to 100	6 to 100			
Median	56	53			
PSG Parameters					
AHI, /h, mean ± SD	40.75 ± 21.38	9.81 ± 6.73	30.94	25.21 to 36.67	<0.001
RDI, /h, mean ± SD	42.91 ± 20.51	10.02 ± 7.14	32.89	27.34 to 38.44	<0.001
ODI, /h, mean ± SD	30.04 ± 20.82	4.24 ± 1.55	25.80	20.46 to 31.14	<0.001
LSaO_2_, %, mean ± SD	81.40 ± 8.18	90.75 ± 1.91	9.35	7.20 to 11.50	<0.011

CI, confidence interval; CFS, Chalder fatigue scale; SD, standard deviation; DHS-R, daily hassles scale revised; CD-RISC, Connor–Davidson resilience scale; PSG, polysomnography; AHI, apnea–hypopnea index; RDI, respiratory distress index; ODI, oxygen desaturation index; and LSaO_2_, lowest oxygen saturation.

**Table 4:** Differences in the CFS, DHS-R, and CD-RISC scores between the obstructive sleep apnea group and the control group 6 months after surgery.

	Postoperative OSA Group (*n* = 60)	Control Group (*n* = 32)	Mean Difference	95% CI	*p*-Value
CFS score, mean ± SD	25.32 ± 7.34	23.97 ± 5.24	1.35	−1.56 to 4.26	0.107
Range	14 to 53	19 to 38			
Median	25	28			
DHS-R score, mean ± SD	70.67 ± 18.25	68.72 ± 15.43	1.95	−5.59 to 9.49	0.219
Range	51 to 150	51 to 111			
Median	71	80			
CD-RISC score, mean ± SD	72.14 ± 19.41	72.25 ± 13.01	0.11	−7.49 to 7.71	0.426
Range	6 to 100	45 to 100			
Median	53	72			
PSG parameters					
AHI, /h, mean ± SD	9.81 ± 6.73	1.28 ± 0.98	8.53	6.15 to 10.91	0.023
RDI, /h, mean ± SD	10.02 ± 7.14	2.30 ± 1.51	7.72	5.18 to 10.26	0.017
ODI, /h, mean ± SD	4.24 ± 1.55	1.46 ± 1.22	2.78	2.15 to 3.41	<0.001
LSaO_2_, %, mean ± SD	90.75 ± 1.91	91.75 ± 2.70	1.00	0.04 to 1.96	0.047

OSA, obstructive sleep apnea; CI, confidence interval; CFS, Chalder fatigue scale; SD, standard deviation; DHS-R, daily hassles scale revised; CD-RISC, Connor–Davidson resilience scale; PSG, polysomnography; AHI: apnea–hypopnea index; RDI: respiratory distress index; ODI: oxygen desaturation index; and LsaO_2_: lowest oxygen saturation.

**Table 5:** Differences in the CFS, DHS-R and CD-RISC scores between the surgical success group and control group 6 months after surgery.

	Success Group (*n* = 43)	Control Group (*n* = 32)	Mean Difference	95% CI	*p*-Value
CFS score, mean ± SD	24.67 ± 1.48	23.97 ± 5.24	0.70	−0.97 to 2.37	0.265
Range	14 to 42	19 to 38			
Median	26	28			
DHS-R score, mean ± SD	69.54 ± 16.77	68.72 ± 15.43	0.82	−6.72 to 8.36	0.186
Range	51 to 120	51 to 111			
Median	75	80			
CD-RISC score, mean ± SD	73.43 ± 21.55	72.25 ± 13.01	1.18	−7.39 to 9.75	0.103
Range	34 to 100	45 to 100			
Median	53	72			
PSG parameters					
AHI, /h, mean ± SD	2.36 ± 1.44	1.28 ± 0.98	1.08	0.49 to 1.67	0.039
RDI, /h, mean ± SD	4.52 ± 2.08	2.30 ± 1.51	2.22	1.35 to 3.09	0.046
ODI, /h, mean ± SD	3.77 ± 1.97	1.46 ± 1.22	2.31	1.52 to 3.10	0.044
LSaO_2_, %, mean ± SD	90.99 ± 1.48	91.75 ± 2.70	0.76	−0.21 to 1.73	0.176

OSA: obstructive sleep apnea; CI: confidence interval; CFS: Chalder fatigue scale; SD: standard deviation; DHS-R: daily hassles scale revised; CD-RISC: Connor–Davidson resilience scale; PSG: polysomnography; AHI: apnea–hypopnea index; RDI: respiratory distress index; ODI: oxygen desaturation index, and LsaO_2_: lowest oxygen saturation.

**Table 6:** Differences in the CFS, DHS-R, and CD-RISC scores between the surgical failure group and control group 6 months after surgery.

	Failure Group (*n* = 17)	Control Group (*n* = 32)	Mean Difference	95% CI	*p*-Value
CFS score, mean ± SD	26.45 ± 6.61	23.97 ± 5.24	2.48	−0.99 to 5.95	0.361
Range	14 to 53	19 to 38			
Median	27	28			
DHS-R score, mean ± SD	71.90 ± 18.21	68.72 ± 15.43	3.18	−6.74 to 13.10	0.202
Range	51 to 150	51 to 111			
Median	81	80			
CD-RISC score, mean ± SD	69.42 ± 19.30	72.25 ± 13.01	2.83	−6.49 to 12.15	0.188
Range	6 to 100	45 to 100			
Median	55	72			
PSG parameters					
AHI, /h, mean ± SD	28.72 ± 4.15	1.28 ± 0.98	27.44	25.90 to 28.98	<0.001
RDI, /h, mean ± SD	30.10 ± 5.52	2.30 ± 1.51	27.80	25.72 to 29.88	<0.001
ODI, /h, mean ± SD	15.77 ± 7.98	1.46 ± 1.22	14.31	11.44 to 17.18	<0.001
LSaO_2_, %, mean ± SD	85.80 ± 4.32	91.75 ± 2.70	5.95	3.93 to 7.97	<0.001

OSA: obstructive sleep apnea; CI: confidence interval; CFS: Chalder fatigue scale; SD: standard deviation; DHS-R: daily hassles scale revised; CD-RISC: Connor–Davidson resilience scale; PSG: polysomnography; AHI: apnea–hypopnea index; RDI: respiratory distress index; ODI: oxygen desaturation index; and LSaO_2_: lowest oxygen saturation.

## Data Availability

Our data is unavailable due to privacy or ethical restrictions.
